# Differences in Stress-Induced Modulation of the Auditory System Between Wistar and Lewis Rats

**DOI:** 10.3389/fnins.2018.00828

**Published:** 2018-11-19

**Authors:** Agnieszka J. Szczepek, Gunnar P. H. Dietz, Uta Reich, Olga Hegend, Heidi Olze, Birgit Mazurek

**Affiliations:** ^1^Department of Otorhinolaryngology, Head and Neck Surgery, Berlin Institute of Health, Charité-Universitätsmedizin Berlin, Corporate Member of Freie Universität Berlin, Humboldt-Universität zu Berlin, Berlin, Germany; ^2^Department of Medicinal Sciences, Dr. Willmar Schwabe GmbH & Co., KG, Ettlingen, Germany; ^3^Tinnitus Center, Berlin Institute of Health, Charité-Universitätsmedizin Berlin, Corporate Member of Freie Universität Berlin, Humboldt-Universität zu Berlin, Berlin, Germany

**Keywords:** auditory perception, auditory threshold, DPOAE, auditory brainstem response, stress, psychological

## Abstract

Many aspects of stress-induced physiological and psychological effects have been characterized in people and animals. However, stress effects on the auditory system are less explored and their mechanisms are not well-understood, in spite of its relevance for a variety of diseases, including tinnitus. To expedite further research of stress-induced changes in the auditory system, here we compare the reactions to stress among Wistar and Lewis rats. The animals were stressed for 24 h, and subsequently we tested the functionality of the outer hair cells (OHCs) using distortion product otoacoustic emissions (DPOAEs) and auditory neurons using evoked auditory brainstem responses (ABR). Lastly, using Western blot, we analyzed the levels of plasticity-related proteins in the inferior colliculus, confirming that the inferior colliculus is involved in the adaptive changes that occur in the auditory system upon stress exposure. Surprisingly, the two strains reacted to stress quite differently: Lewis rats displayed a lowering of their auditory threshold, whereas it was increased in Wistar rats. These functional differences were seen in OHCs of the apical region (low frequencies) and in the auditory neurons (across several frequencies) from day 1 until 2 weeks after the experimental stress ended. Wistar and Lewis rats may thus provide models for auditory threshold increase and decrease, respectively, which can both be observed in different patients in response to stress.

## Introduction

Emotional stress can be defined as a mental tension resulting from a challenging situation. Short-term stress enables adaptation to the new environment and is generally of evolutionary advantage. Long-term stress, however, may have detrimental consequences on the nervous system (Musazzi et al., 2017), the reproductive system (Arck et al., 1995; Arck, 2001), and many other organ functions. Stress is acting on the affected persons or animals *via* the hormonal axes (e.g., hypothalamus-pituitary-adrenal, HPA), via the neuronal axis of the sympathetic nervous system, and *via* changes in the secondary target tissues, such as the immune system. The HPA response is known to occur relatively quickly after exposure to stress and can be monitored by measuring the concentration of cortisol (in people) or corticosterone (in rats), which usually increases within minutes after stress exposure. In contrast, the response of immune system is often delayed. It can be monitored by measuring the concentration of proinflammatory cytokines such as TNFα, and occurs within hours to days after stress exposure.

The consequences of long term stress may be detrimental and include an increased risk for heart attack and stroke (Kornerup et al., 2010), progression of inflammatory bowel diseases (Ananthakrishnan, 2015), and many other diseases such as psoriasis or rheumatoid arthritis (Rampton, 2011).

Stress also has consequences for the auditory perception. In people, stress has been implicated to improve auditory attention (Hoskin et al., 2014b) and to stimulate the somatosensory part of the brain (Bierzynska et al., 2015), possibly contributing to auditory hallucinations (Hoskin et al., 2014a). Emotional stress is also known to associate with various hearing conditions such as sudden hearing loss, Meniere's disease, tinnitus, or hyperacusis (Rauschecker et al., 2010, 2015; Mazurek et al., 2012b). Moreover, acute stress increases auditory reaction times in healthy young people (Pradhan et al., 2014) and in healthy and autistic children (Fujikawa-Brooks et al., 2010), suggesting close interactions between stress response and cognitive function.

There are various experimental models of stress to study its consequences at the molecular and cellular level. These models involve physical- or psycho-social stressors (Mazurek et al., 2012b). Stressors include electric shock or restraining the animal, or the exposure to a scent of a predator, or solitary confinement. Some other models try to reproduce aspects of stress occurring in the human environment, such as long-term exposure to sound that is unpleasant but not loud enough to damage the auditory system. Such animal models using sonic stress for e.g., 24 h have been used to demonstrate deleterious effects of stress on pregnancy (Arck et al., 1995), hair loss (Arck et al., 2003), or asthma (Joachim et al., 2004), to mention only a few.

Part of basic research on emotional stress has been dedicated to determine how stress contributes to mood disorders or to anxiety. Interestingly, it has been demonstrated that within the same species (e.g., rats), various strains may react differently to the same stressor. In an experimental stroke model, depression-like symptoms were observed in Lewis but not in Wistar rats (Kunze et al., 2014). Moreover, different strains of rats reacted differentially to an identical stressor, with insomnia being the outcome measurement (Tang et al., 2005). The different reactions of Wistar and Lewis rats to stress may depend on various reactivity in the HPA axis (HPAa); Lewis being hypo-responsive to stress due to higher amounts of corticotropin releasing hormone in the hypothalamus, as compared to other strains (Calogero et al., 1992). Further differences were observed between Lewis and Wistar rats in a model for obsessive-compulsiveness, in which Lewis rats were predisposed to develop the disease, while Wistar rats were resistant to it (Brimberg et al., 2007).

Pioneering experiments performed in the seventies and eighties demonstrated that restraint stress can affect the hearing thresholds of guinea pigs (Muchnik et al., 1980) and induce hearing loss. Interestingly, the auditory reaction was noted only in 50% of experimental animals exposed to stress. In another set of experiments, neuronal atrophy in the inferior colliculus was demonstrated to occur in Sprague-Dawley rats daily exposed to 2 h restraint stress for 10 days (Dagnino-Subiabre et al., 2005). In our earlier work, we found that following stress, the hearing threshold of Wistar rats changes and that the animals hear better at all frequencies. The changes were temporary and lasted < 1 week (Mazurek et al., 2010). In addition, we found temporary changes in gene expression occurring along the auditory pathway of stressed animals (Mazurek et al., 2010, 2012a).

Because the behavioral responses to stress differ between various strains of rats, and because stress changes the physiological performance and gene-expression of the auditory system, we hypothesized that the auditory answer to stress may vary, depending on the strain. Thus, different rat strains could be selected as models for stress-induced auditory changes, depending on the question to be addressed. To test this hypothesis, we have exposed Wistar and Lewis rats to a stress paradigm previously described (Mazurek et al., 2010). Next, we have analyzed the auditory responses of animals and compared the audiometric changes as well as modulation of protein levels occurring in the inferior colliculus and in serum after stress.

## Materials and methods

### The animals

This study was carried out in accordance with the recommendations of the EU Directive 2010/63/EU on the protection of animals used for scientific purposes. The Governmental Ethics Commission for Animal Welfare approved the experimental protocol (LaGeSo Berlin, Germany; approval number: G 0255/12). The experimental distribution of animals is presented in Table 1. In total, 80 animals were used.

**Table 1:** Number of animals analyzed under control conditions or at the different time points after stress.

	**ABR (# of animals used)**	**ABR (# of ears tested)**	**DPOAE (# of animals used)**	**DPOAE (# of ears tested)**
**LEWIS**				
Control	6	11	7	7
Immediately after stress	10	13	10	17
One day after stress	10	13	10	17
One week after stress	10	16	10	17
Two weeks after stress	11	16	11	21
Total	47	69	48	79
**WISTAR**				
Control	4	7	4	8
Immediately after stress	8	12	8	14
One day after stress	6	8	6	10
One week after stress	7	10	7	14
Two weeks after stress	7	10	7	14
Total	32	47	32	60

**Figure 1:**
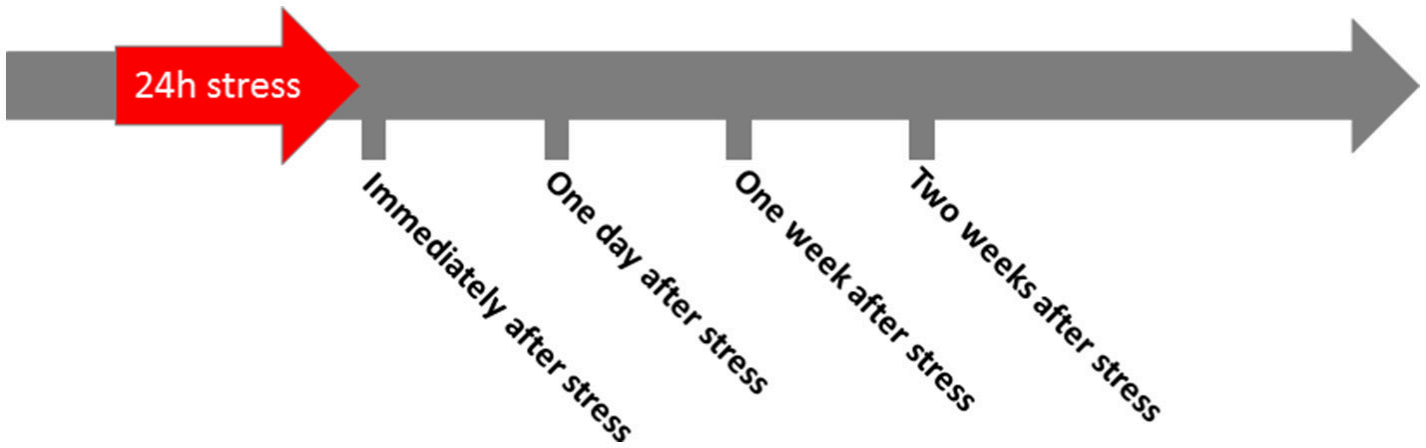
Schematic presentation of the experimental design. Immediately after stress, 24 h after stress, 1 week, or 2 weeks after stress electrophysiological measurements were performed, the rats were sacrificed, and blood and brain tissue was collected.

Wistar (Crl:WI) and Lewis (LEW/Crl) female rats were purchased from Charles River Laboratories (Sulzfeld, Germany). All animals were 4 weeks old at delivery. Upon arrival, the animals were transferred to their home cages (2–4 animals per cage) and left there for 7 days to adapt to the new environment. The animal facility provides 12/12 h light/darkness conditions with a standard chow and water *ad libitum*. After the adaptation time, the animals were subjected to experimental stress for 24 h (Figure 1), by exposing them to a rodent repeller (Conrad Electronics, Berlin, Germany) producing sound of low frequency (300–350 Hz) and low intensity (61–65 dB SPL) and the respective vibrations (Mazurek et al., 2010). In order to prevent additional anxiety during the stress period, always two animals were kept in one cage without enrichment (Campos et al., 2013).

After stress exposure for 24 h, the animals were either immediately transferred to the laboratory for audiometric testing or they were left under non-stress conditions in the animal facility for 1, 7, or 14 days. All experiments were performed at the same time of the day. The animals were anesthetized between 8:00 and 8:30 a.m., the audiometric measurements were performed immediately afterwards and took on average till 11:00 a.m., when the animals were sacrificed.

In preparation for the audiometric measurements, the animals were anesthetized with ketamine/xylazine (100 mg ketamine/kg and 5 mg xylazine/kg, i.m.). Next, the audiometric measurements were performed on an anti-vibration table in a noise-controlled environment of a soundproof chamber. The stimulation signals were generated by the TDT System3 hardware containing RX6 multifunction processor, PA5 programmable attenuator, ED1 electrostatic speaker driver, EC1 loud speaker, RA4PA 4-channel preamplifier, and RA4LI-4-channel head stage (Tucker-Davis Technologies, USA).

### Auditory brainstem responses (ABR)

Disposable subdermal needle electrodes (Rochester Electro-Medical, Inc., Nederlands) 12 mm long, 27 Ga were used for auditory brainstem response (ABR) measurements. The active electrode was placed on the vertex and the reference electrode was placed on the mastoid, the ground electrode was placed in the leg of the anesthetized animal. To evoke the ABR signals in the auditory system of the anesthetized rat, the pure tone signals were applied in the frequencies 500 Hz, 1, 2, 4, 8, 16, and 32 kHz and were presented from the sound intensity of 65–20 dB in 5 dB steps intervals for each frequency. The ABR signal was amplified (gain 20-fold, sampling rate: 25 kHz) and analyzed using a real-time processor in the TDT system3. The ABR signals were averaged using filter settings (high pass: 300 Hz; low pass: 3 kHz). The average of 1,000 ABR waveforms (duration 10 ms) was displayed on a PC monitor during the experiments using operating software (BioSigRP, TDT). The hearing threshold was defined for each frequency by the amplitude value of the last detectable ABR wave i.e., the lowest value reproducing responses of the most prominent ABR wave (i.e., wave III of the early acoustic evoked potentials).

### Distorted product otoacoustic emissions (DPOAE)

The sound stimulus consisted of simultaneous permanent pure tones at two different frequencies (f2/f1 ratio = 1.22) between 60 and 25 dB (L1 = L2) in 5 dB steps (Kemp, 2002). Distortion product otoacoustic emissions (DPOAEs) were measured at five frequencies: 2, 4, 8, 12, 16, and 32 kHz. The DPOAE signals were amplified and averaged (100 waveforms, duration 168 ms) and displayed using TDT software. The hearing thresholds for each frequency were defined as a numerical value of the last detectable amplitude that was produced in response to the acoustic stimulation.

### Preparation of the auditory brain

After completing the audiometric analyses, a lethal dose of ketamine/xylazine was injected i.m. and the blood was collected from the carotid artery. The brain was removed immediately after the animal's death and the inferior colliculi were isolated under a stereoscope (ZEISS, Jena, Germany), and tissue suspended in a lysis RTL buffer (cat.# 79216, Qiagen, Hilden, Germany) and stored at −80°C.

### Western blot

The concentration of protein was measured using the micro BCA protein assay (Life Technologies GmbH, Darmstadt, Germany, cat. # 23235). Aliquots containing 10 μg of total protein were mixed with Roti-Load sample loading solution (ROTH #K929.1) and heated at 90°C for 5 min in a Thermomixer comfort (Eppendorf Vertrieb Deutschland GmbH, Wesseling-Berzdorf, Germany) loaded onto Novex WedgeWell 4–20% Tris-Glycine Mini Gels, 12 well (Thermo Fischer Scientific, Schwerte, Germany, cat. # XP04202BOX) or 15-well gradient gels Novex WedgeWell 4–20% Tris-Glycine Mini Gels (Thermo Fischer Scientific, cat. # XP04205BOX), and resolved using mini-SDS-PAGE system XCell SureLock Electrophoresis Cell (Invitrogen, cat. # 1287724-0959) at 130 V for 1 h and 40 min. Protein marker used was a PageRuler Plus Prestained Protein Ladder (Thermo Fischer Scientific, cat. # 26619).

After electrophoresis, resolved proteins were transferred onto 0.45 μm Immobilon-P Transfer Membrane (Millipore, cat. # IPFL 000 10) using XCell II Blot Module (Invitrogen, cat. # EI9051) at 300 mA for 44 min (power supply Biometra GmbH, Göttingen, Germany). The membranes were then blocked with 5% skimmed milk powder solution prepared in phosphate-buffered saline (PBS) and containing 0.05%Tween 20 (Sigma-Aldrich) for 1 h at room temperature.

The membranes were incubated for 2 h at room temperature with one of the primary antibodies (see Table 2). Following a triple wash in PBS 0.05%Tween 20 for 10 min each, secondary antibodies were added, consistent with the primary one used. The secondary antibodies included goat-anti-rabbit IgG (H+L), HRP conjugate (Promega GmbH, Mannheim, Germany, cat. # W4011), and goat-anti-mouse IgG (H+L), HRP conjugate (Promega GmbH, cat. # W4021). The reaction was developed by adding SuperSignal West Femto Maximum Sensitivity Substrate (Thermo Fischer Scientific cat. # 34095). Chemiluminescence was captured directly and measured by C-Digit blot scanner (LI-COR Biotechnology—GmbH, Bad Homburg, Germany).

**Table 2:** Antibodies used in Western blots.

**Target**	**Host**	**Product size (kDa)**	**Manufacturer**	**Catalog number**	**Dilution**
AMPA receptor	Rabbit	100	Cell Signaling	2460S	1:500
Arc	Mouse	55	Thermo Scientific	PA1-30682	1:500
Syt1	Rabbit	60	Cell Signaling	3347	1:1,000
Syt12	Rabbit	47	Sigma-Aldrich	HPA011006	1:1,000
ß-Actin	Mouse	42	Sigma-Aldrich	A5441	1:10,000

### Blood collection and processing

Between 500 and 900 μl of blood was collected from animals at the end of the experiment *via* exsanguination from the carotid artery (for all animals at 10 a.m. ± 10 min). The blood was allowed to coagulate for 30 min at room temperature in a 1.5 ml Eppendorf tube, followed by centrifugation at 14,000 rpm in the Eppendorf centrifuge and collection of serum. Serum was transferred into clean tube and immediately stored at −80°C in 100 μl aliquots.

### Enzyme-linked immunosorbent assay (ELISA)

We performed ELISA to measure the concentration of corticosterone and tumor necrosis factor alpha (TNF-alpha) in sera of animals; the corresponding assay systems were purchased from Abnova (Corticosterone ELISA Kit, Abnova GmbH, Heidleberg, Germany, cat. # B0AP01090J00015) and Thermo Fischer Scientific (Rat TNF-alpha ELISA Kit, cat. # ER3TNFA), respectively. Samples were assayed in duplicates adhering strictly to manufacturer directions. Optical density of samples on the 96 well ELISA plate was measured using Spectramax M2 (Molecular Devices, Sunnyvale, CA, USA) and computed with SoftMax Pro, V5 software (Molecular Devices).

### Statistical analyses

Statistical analyses were performed using SigmaPlot V12 software. First, we tested for the type of distribution. For normally distributed samples, the *t*-test was performed; the rest were tested using the Mann-Whiney-*U* Test. Both tests were two-sided; the alpha value was set to 0.05 in both tests.

The average control threshold (baseline threshold) was calculated for each frequency and strain using the data obtained from unstressed control animals. Next, the hearing threshold values obtained from stressed animals were subtracted from the baseline. The values below zero indicate elevated hearing threshold, signifying impaired hearing abilities as compared to the controls. The values above zero indicate lowered hearing threshold, signifying improved hearing abilities as compared to the controls. At the end of each Figure legend, we have also added information on the statistical method applied.

## Results

### At baseline, wistar and lewis rats display similar hearing thresholds

**Figure 2:**
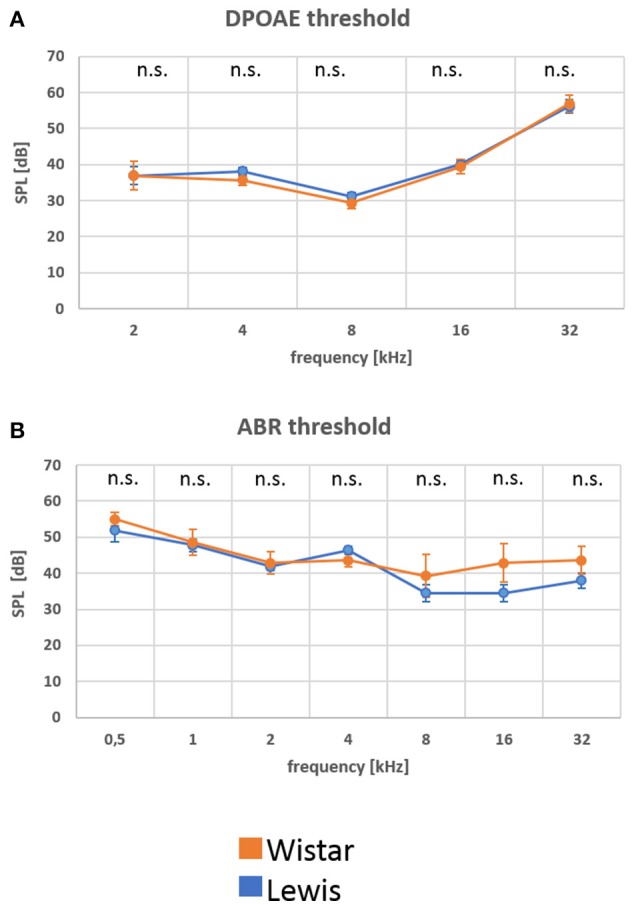
**(A,B)** At baseline, Wistar and Lewis rats display similar electrophysiological characteristics in the auditory system. Distortion product otoacoustic emissions (DPOAE) were measured by stimulating the ear with two different frequencies and by determining the sound threshold that rendered a detectable sound. To detect changes in the function of the inner ear, the acoustic nerve and the auditor brainstem, auditory brainstem responses (ABR) were measured using subdermal electrodes and stimulation at the frequencies indicated. Nine Wistar rats (17 measurements of ABR and 18 measurements of DPOAE) and 13 Lewis rats (22 measurements of ABR and 24 measurements of DPOAE) were used for these baseline measurements. Presented are means for each frequency with standard deviations. The significance of differences between the two strains was tested using the Mann-Whitney Rank Sum Test; the alpha value was set to 0.05.

Aiming to determine possible differences in basic hearing function between control, non-stressed Wistar or Lewis rats, we analyzed hearing in young adult (2–3 months old) rats of each strain. The function of the outer hair cell (OHC) was measured by determining DPOAE thresholds at frequencies between 2 and 32 kHz. These measurements indicated no significant differences in the DPOAE values for neither one of the frequencies tested (Figure 2A; number of animals given in Table 1), suggesting that there were no strain differences at the level of the hair cell function among Wistar and Lewis rats. Hearing function at the next level was examined by determining click stimulus-evoked ABR thresholds (Figure 2B number of animals given in Table 1). ABR to click stimuli confirmed no significantly elevated average auditory threshold between the two rat strains at any of the tested frequencies. A tendency toward a slightly higher sensitivity of Lewis rats at 16 and 32 kHz was not statistically significant. These results are consistent with the assumption that unstressed Wistar and Lewis rats do not differ in their baseline hearing.

Having characterized the animals at baseline, we next examined how the two rat strains reacted to stress.

### Blood stress-related proteins differ among rat strains after stimulation

**Figure 3:**
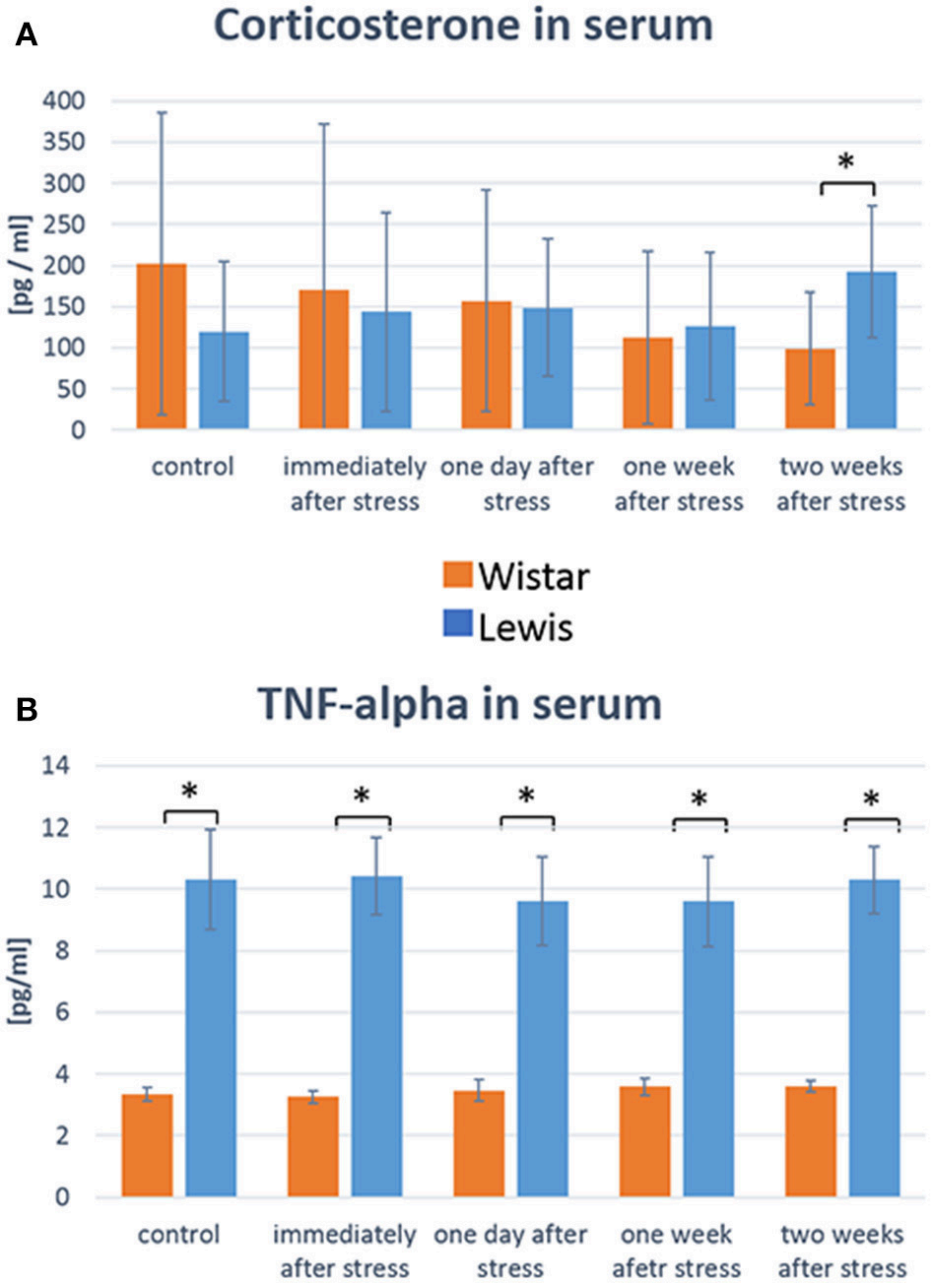
Wistar and Lewis rats differ in their concentration of stress-related molecules in serum. Concentrations of corticosterone **(A)** and TNF-alpha **(B)** were measured in the serum of Wistar (*n* = 54) and Lewis (*N* = 37) rats using commercially available ELISA kits. Number of serum samples from Wistar rats used for measurement: control *n* = 9, immediately after stress *n* = 8, 1 day after stress *n* = 6, 1 week after stress *n* = 7, 2 weeks after stress *n* = 7. Number of serum samples from Lewis rats used for measurement: control *n* = 13, immediately after stress *n* = 10, 1 day after stress *n* = 10, 1 week after stress *n* = 10, 2 weeks after stress *n* = 11. Significant differences between Lewis and Wistar rats calculated with Mann-Whitney Rank Sum Test were marked with asterisks indicating two-tailed *P* < 0.05.

As our overall goal was to characterize the effect of stress specifically on the auditory system, we needed to assess whether both rat strains were generally affected by stress in a similar way at the time of measurements. A well-established approach to do so is to measure plasma levels of markers known to be elevated in response to stress, such as glucocorticoids or inflammatory cytokines. We thus determined serum levels of corticosterone and TNFα at those time points after stress when we also determined auditory function. The mean values of corticosterone tended to drop in Wistar rats up to 14 days after stress compared to baseline control, while they tended to increase in Lewis rats 14 days after the end of the stress period (Figure 3A). While due to a high inter-individual variability, that change did not reach statistical significance, a significant difference between the higher corticosterone serum level in Lewis rats compared to Wistar rats could be detected at the 2 weeks time-point, suggesting that both strains differently respond to and recover from stress stimuli (Koolhaas et al., 1999). Consistent with that assumption, TNF-alpha levels were also different between Wistar and Lewis rat, at baseline and up to 2 weeks after stress (Figure 3B). Next, we sought to examine whether those strain-specific differences in stress related endocrine regulation correlated with differential stress related adaptations in the auditory system.

### Auditory function adapts differently to stress in wistar and lewis rats

**Figure 4:**
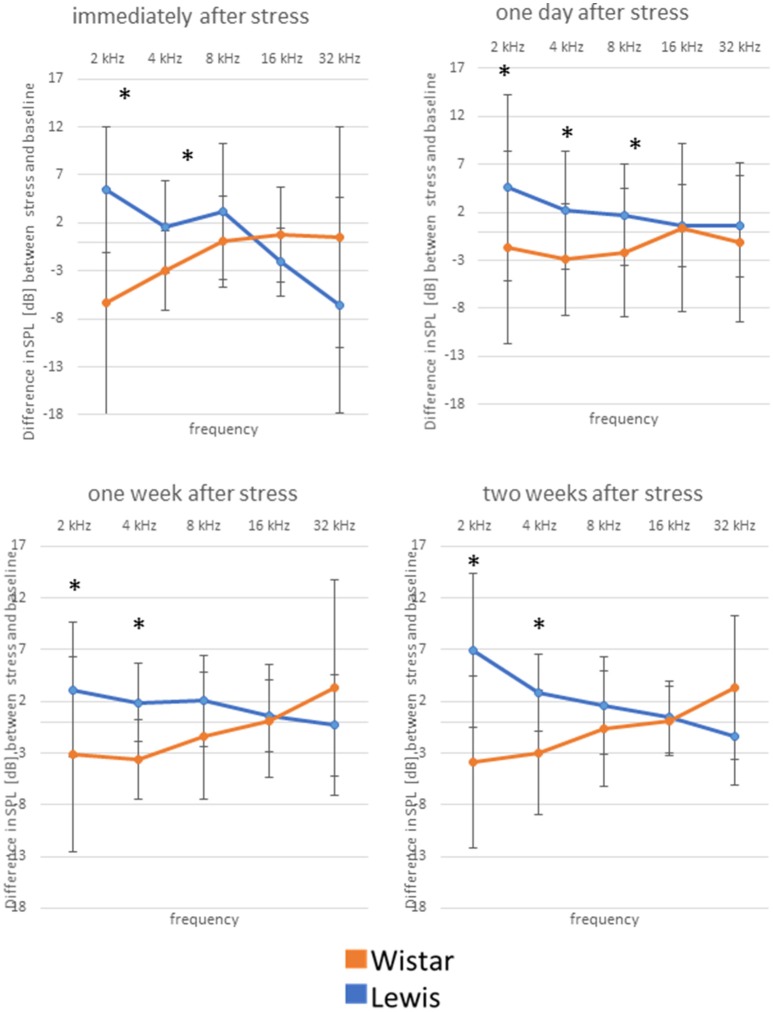
Wistar and Lewis rats cochlear hair cells adjust differently to stress. The animals were stressed for 24 h, and DPOAEs were measured immediately after the exposure (upper left); 1 day after stress (upper right); 1 week after stress (lower left); or 2 weeks after (lower right) exposure. Shown are DPOAE changes from baseline. Presented are the mean differences from control, not stressed animals Blue lines, Lewis rats; orange lines, Wistar rats. Number of ears used for DPOAE measurement: control *n* = 9, immediately after stress *n* = 8, 1 day after stress *n* = 6, 1 week after stress *n* = 7, 2 weeks after stress *n* = 7. Number of Lewis rats used for DPOAE measurement: control *n* = 13, immediately after stress *n* = 10, 1 day after stress *n* = 10, 1 week after stress *n* = 10, 2 weeks after stress *n* = 11. The values below zero indicate elevated hearing thresholds, signifying impaired hearing abilities as compared to the controls. The values above zero indicate lowered hearing threshold, signifying improved hearing abilities as compared to the controls. Significant differences between Lewis and Wistar rats calculated with Mann-Whitney Rank Sum Test were marked with asterisks indicating two-tailed *P* < 0.001.

The difference in stress-related hormones and previous observations (Oitzl et al., 1995) led us to hypothesize that the auditory system of the two rat strains could respond to stress disparately. Indeed, we found that auditory pathways of Wistar and Lewis rats reacted in strain-specific opposite ways to the 24 h stress. DPOAE measurements indicated that at lower frequencies (2 and 4 kHz—for statistics see Table 3), Wistar rats adopted higher hearing thresholds compared to Lewis rats, indicating decreased hearing abilities as compared to the non-stressed (both Lewis and Wistar) and stressed Lewis rats at all time-points measured (Figure 4, see for statistics Table 3). In addition, 1 day after stress, Lewis rats had significantly increased hearing abilities also at 8 kHz, as compared to Wistar rats, demonstrating that already at the level of the OHC, Wistar and Lewis auditory systems react to stress differently.

**Table 3:** Stress-induced differences in DPOAE between Lewis and Wistar rats.

**Group**	***N***	**Median**	**25%**	**75%**
**A. IMMEDIATELY AFTER STRESS**				
**2 kHz**				
Lewis	17	6.923	1.923	11.923
Wistar	14	−3.125	−14.375	1.875
Mann-Whitney-*U*-Test (two-tailed)			*p* < 0.001	
*U*-value:	30			
**4 kHz**				
Lewis	17	3.077	−1.923	5.000
Wistar	14	−4.375	−4.375	0.625
Mann-Whitney-*U*-Test (two-tailed)			*p* = 0.001	
*U*-Value:	51			
**B. ONE DAY AFTER STRESS**				
**2 kHz**				
Lewis	17	6.923	−0.577	11.923
Wistar	10	−0.625	−6.875	6.875
Mann-Whitney-*U*-Test (two-tailed)			*p* = 0.018	
*U*-Value:	38			
**4 kHz**				
Lewis	17	3.077	−4.423	8.077
Wistar	10	−4.375	−9.375	1.875
Mann-Whitney-*U*-Test (two-tailed)			*p* = 0.050	
*U*-Value:	46		
**8 kHz**				
Lewis	17	1.154	−1.346	6.154
Wistar	10	−0.625	−6.875	4.375
Mann-Whitney-*U*-Test (two-tailed)			*p* = 0.043	
*U*-Value:	45			
**C. ONE WEEK AFTER STRESS**				
**2 kHz**				
Lewis	17	6.923	−3.077	6.923
Wistar	14	−3.125	−9.375	3.125
Mann-Whitney-*U*-Test (two-tailed)			*p* = 0.009	
*U*-Value:	53		
**4 kHz**				
Lewis	17	3.077	−1.923	3.077
Wistar	14	−4.375	−5.625	0.625
Mann-Whitney-*U*-Test (two-tailed)			*p* = 0.001	
*U*-Value:	31		
**D. TWO WEEKS AFTER STRESS**				
**2 kHz**				
Lewis	21	6.923	6.923	11.923
Wistar	14	−3.125	−13.125	3.125
Mann-Whitney-*U*-Test (two-tailed)			*p* < 0.001	
*U*-Value:	25		
**4 kHz**				
Lewis	21	3.077	3.077	3.077
Wistar	14	−1.875	−4.375	0.625
Mann-Whitney-*U*-Test (two-tailed)			*p* < 0.001	
*U*-Value:	43		

*Only significantly different values are shown for measurements immediately after stress (A); 1 d after stress (B); 1 week after stress (C); 2 weeks after stress (D). Significance was determined applying the Mann-Whitney Rank Sum Test. Values are given as the median, with 25 and 75% confidence intervals provided*.

**Figure 5:**
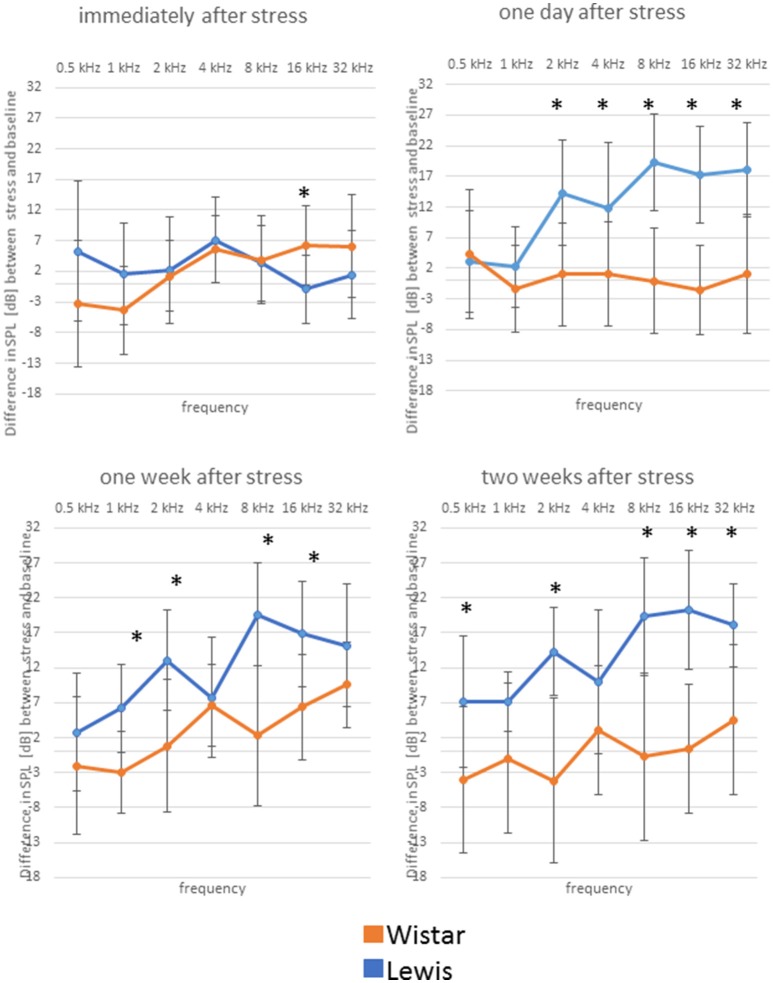
Delayed improved hearing abilities in Lewis rats, but not in Wistar rats after stress exposure. Shown are changes from baseline of ABR measured immediately after stress exposure; 1 day after stress; 1 week after stress; or 2 weeks after stress; as indicated above the individual graphs, presented as the mean difference to the ABR in control animals. Blue lines, Lewis rats; orange lines, Wistar rats. Number of Wistar rats used for ABR measurement: control *n* = 9, immediately after stress *n* = 8, 1 day after stress *n* = 6, 1 week after stress *n* = 7, 2 weeks after stress *n* = 7. Number of ears used for ABR measurement: control *n* = 13, immediately after stress *n* = 10, 1 day after stress *n* = 10, 1 week after stress *n* = 10, 2 weeks after stress *n* = 11. The values below zero indicate elevated hearing threshold, signifying impaired hearing abilities as compared to the controls. The values above zero indicate lowered hearing threshold, signifying improved hearing abilities as compared to the controls. Significant differences between Lewis and Wistar rats, calculated with Mann-Whitney Rank Sum Test, were marked with asterisks indicating two-tailed *p* < 0.001.

We next determined how higher auditory function was affected in the two strains by assessing the post-stress ABR levels. Immediately after stress, significant differences between the two rat stains were found for 16 kHz, with Wistar rats performing better than the control, while Lewis rats did not show changes compared to baseline. One day after finishing stress, hearing abilities of Wistar rats were poorer compared to these of Lewis rats at the frequencies of 2, 4, 8, 16, and 32 kHz. One week after finishing the stress period, the high frequencies of Lewis rats were still positively affected by stress with significant differences at 1, 2, 8, and 16 kHz, hearing about 17 dB better when compared to baseline and stressed Wistar rats. Two weeks after finishing stress, Lewis rats hearing abilities were still significantly better than baseline in the frequencies of 0.5, 2, 8, 16, and 32 kHz (Figure 5, see for statistics Table 4).

**Table 4:** Stress-induced differences in ABR between Lewis and Wistar rats.

**Group**	***N***	**Median**	**25%**	**75%**
**A. IMMEDIATELY AFTER STRESS**				
**16 kHz**				
Lewis	13	−0.500	−5.500	4.500
Wistar	12	5.357	2.857	11.607
Mann-Whitney-*U*-Test (two-tailed)			*p* = 0.020	
*U*-Value:	35			
			**std Dev**	**SEM**
**B. ONE DAY AFTER STRESS**				
**2 kHz**				
Lewis	12	14.318	8.660	2.500
Wistar	8	0.982	8.425	2.979
*t*-test (two-tailed)			*p* = 0.00313	
Confidence interval for difference of means:			5.118–21.554	
**4 kHz**				
Lewis	12	11.818	10.660	3.077
Wistar	8	1.071	8.452	2.988
*t*-test (two-tailed)			*p* = 0.0281	
Confidence interval for difference of means:			1.292–20.202	
**8 kHz**				
Lewis	12	19.318	7.833	2.261
Wistar	8	−0.0893	8.634	3.053
*t*-test (two-tailed)			*p* < 0.0001	
Confidence interval for difference of means:			11.588–27.227	
**16 kHz**				
			**25%**	**75%**
Lewis	12	16.818	8.068	25.568
Wistar	8	0.357	−9.643	2.857
Mann-Whitney-*U*-Test (two-tailed)			*P* < 0.001	
*U*-Value:	3			
**32 kHz**				
			**std Dev**	**SEM**
Lewis	12	18.068	7.724	2.230
Wistar	8	1.071	9.636	3.407
*t*-test (two-tailed)			*p* = 0.0004	
Confidence interval for difference of means:			8.828–25.166	
**C. ONE WEEK AFTER STRESS**				
**1 kHz**				
Lewis	16	6.193	6.292	1.573
Wistar	10	2.929	5.798	1.833
*t*-test (two-tailed)			*p* = 0.0011	
Confidence interval for difference of means:			4.038–14.206	
**2 kHz**				
Lewis	16	13.068	7.188	1.797
Wistar	10	0.857	9.487	3.000
*t*-test (two-tailed)			*p* = 0.001	
Confidence interval for difference of means:			5.450–18.972	
**8 kHz**				
Lewis	16	19.631	7.296	1.824
Wistar	10	2.286	10.055	3.180
*t*-test (two-tailed)			*p* < 0.001	
Confidence interval for difference of means:			10.325–24.365	
**16 kHz**				
Lewis	16	16.818	7.528	1.882
Wistar	10	6.357	7.472	2.363
*t*-test (two-tailed)			*p* = 0.002	
Confidence interval for difference of means:			4.215–16.707	
			**std Dev**	**SEM**
**D. TWO WEEKS AFTER STRESS**				
**0.5 kHz**				
Lewis	16	7.131	9.393	2.348
Wistar	10	−4.000	10.488	3.317
*t*-test (two-tailed)			*p* = 0.0096	
Confidence interval for difference of means:			2.962–19.299	
**2 kHz**				
Lewis	16	14.318	6.325	1.581
Wistar	10	−4.143	11.832	3.742
*t*-test (two-tailed)			*p* < 0.001	
Confidence interval for difference of means:			11.137–25.785	
**8 kHz**				
Lewis	16	19.318	8.367	2.092
Wistar	10	−0.714	12.019	3.801
*t*-test (two-tailed)			*p* < 0.001	
Confidence interval for difference of means:			11.800–28.265	
**16 kHz**				
			**25%**	**75%**
Lewis	16	21.818	16.818	26.818
Wistar	10	2.857	−8.393	7.857
Mann-Whitney-*U*-Test (two-tailed)			*p* < 0.001	
*U*-Value:	11			
**32 kHz**				
			**std Dev**	**SEM**
Lewis	16	18.068	5.916	1.479
Wistar	10	4.571	10.750	3.399
*t*-test (two-tailed)			*p* < 0.001	
Confidence interval for difference of means:			6.778–20.215	

*Only significantly different values are shown for measurements immediately after stress (A); 1 d after stress (B); 1 week after stress (C); 2 weeks after stress (D). Significance was determined applying the Mann-Whitney Rank Sum Test. Values are either given as the median, with 25 and 75% confidence intervals provided in cases where the Mann-Whithey-U-Test could be applied; or, in all cases in which the t-test was applied, as the mean, together with standard deviations and standard errors*.

Taken together, in this part of our work, we demonstrated that even though both strains have very similar auditory baseline functions, after the exposure to 24 h experimental stress, Lewis rats increase whereas Wistar decrease their hearing abilities. These changes were affecting hair cells sensitive at low frequencies, and brainstem responses sensitive to mid and high frequency stimulation. To acquire an initial indication of the possible mechanisms of these strains differences in auditory stress adaptation, we next investigated the levels of proteins commonly associated with neuronal plasticity in the inferior colliculus.

### Stress-induced changes in levels of plasticity-related proteins in the inferior colliculus

In our present investigation, we focused on protein composition in the inferior colliculi rather than the auditory cortex, because the inferior colliculus actively contributes to the generation of the evoked ABRs (Land et al., 2016). We reasoned that the functional changes seen in ABR profiles might be linked to changes occurring in AMPA, Arc, Syt1, Syt12, at the protein level, as those are involved in the synaptogenesis. We found significant changes in the levels of most of the tested proteins at certain time points after stress. In detail, AMPA receptors were significantly downregulated in the IC of Lewis (but not Wistar) rats immediately after stress (Table 5). AMPA receptors were significantly upregulated in Wistar (but not Lewis) rats 2 weeks after stress. The levels of additional proteins involved in neuroplasticity were regulated following stress: Arc was significantly downregulated immediately after stress in Wistar rats, whereas in Lewis rats it was upregulated. Two weeks following stress, Arc was upregulated in Wistar but not in Lewis rats. Syt1, calcium-dependent synaptic protein (Tang et al., 2006) was downregulated immediately and 1 week following stress in Wistar rats, whereas in Lewis rats the levels of Syt1 were stable. Moreover, Syt12, which also belongs to the family of synaptic proteins but is calcium independent (Maximov et al., 2007), was dowregulated in Wistar rats immediately after stress whereas in Lewis rats, it was downregulated immediately, 1 week and 2 weeks after stress.

**Table 5:** Protein levels in the inferior colliculus assessed by Western blotting.

		**Immediately after stress**	**One day after stress**	**One week after stress**	**Two weeks after stress**
Wistar	AMPA	1.046	1.089	1.177	1.248*
	ARC	0.847*	1.055	1.014	1.136*
	Syt1	0.893*	0.916	0.921*	0.992
	Syt12	0.908*	0.928	1.056	0.941
Lewis	AMPA	0.675*	1.037	0.937	1.139
	ARC	1.174*	1.090	1.082	1.012
	Syt1	1.046	1.121	1.081	0.991
	Syt12	0.822*	0.954	0.796*	0.897*

*The numbers represent median fold changes in the optical density of specific protein bands calculated against baseline. All values were normalized against beta-actin originating from each protein sample. The number of control samples tested in the Western blot were n = 16 for Wistar and n = 15 for Lewis rats*.

*Significance of changes was calculated using T-test. Asterisks indicate significance of changes (p > 0.05)*.

*Red to yellow scale of color designates decreased levels whereas green scale designates increased levels of proteins*.

Although several of the detected changes in protein levels reached statistical significance, the only slightly more than 1.5-fold difference in regulation between the two strains was detected for AMPA immediately after stress induction. Thus, induction or downregulation of these four genes cannot explain the strain-specific differences in the stress related adaptations of the auditory system.

## Discussion

Here, we present the novel observation about auditory systems of Wistar and Lewis rats reacting to stress in a strain-dependent manner, where the hearing abilities of Lewis rats increased while the hearing abilities of Wistar rats decreased following stress. Moreover, we determined significant differences in corticosterone and TNF-alpha concentrations between the strains before and after stress. Lastly, we examined the levels of synaptic and apoptosis-related proteins in the inferior colliculi and likewise noticed significant differences in the collicular protein composition between Wistar and Lewis strains following stress.

The influence of stress on hearing abilities can be seen in people (Pradhan et al., 2014) and in experimental animals (Muchnik et al., 1980; Dagnino-Subiabre et al., 2005; Mazurek et al., 2010). The majority of findings suggest development of hearing impairment following stressful situations. In our earlier work, we have demonstrated that the exposure of Wistar rats to 24 h experimental stress conditions, known to induce miscarriage in pregnant animals (Arck et al., 1995) or to provoke the development and progression of dermatitis (Pavlovic et al., 2008), significantly increased the hearing abilities of animals, as compared to the controls (Mazurek et al., 2010). Here, we demonstrate that Wistar rats obtained from a different vendor react in an opposite way to identical stress conditions by a *reduction* of hearing abilities. In the recent years, the observation about Wistar rats reacting to stress in variable fashion has been attributed to differences in the breeding colonies available from different vendors (Paré and Kluczynski, 1997; Pecoraro et al., 2006; Theilmann et al., 2016). The novel finding, which we report here, is about the stress-related auditory responses within the same strain of rats but dependent on a different vendor. Such differences were seen in rat strains from different vendors in studies that examined the behavior of rats following stress (Paré and Kluczynski, 1997; Pecoraro et al., 2006; Theilmann et al., 2016). These differences could be a result of epigenetic regulation or a specific genetic drift in the outbred colony. In our earlier work, we purchased Wistar rats from the Research Institute of Experimental Medicine at our home institution. The change in local regulations led to a shutdown of that breeding facility and therefore we then purchased Wistar rats from Charles River Germany. All other elements of our experimental system remained unchanged, emphasizing the important role of origin of the experimental animals used in stress research with auditory read-out.

Another interesting observation we made was that the inner ear and auditory brainstem both react to emotional stress but that their reaction is not tonotopically identical or synchronized. For instance, the dramatic increase in hearing abilities measured by ABR in Lewis rats 1 day after finishing stress (gain of 12–18 dB in the frequencies between 2 and 32 kHz) is not matched by a similar increase of DPOAEs. It is tempting to speculate that the emotional stress has greater impact on the auditory brainstem or brain than it does on the inner ear.

Differences in the stress response of outbred Wistar rats and inbred Lewis rats have been reported earlier. For instance, Wistar rats were more susceptible to chronic stress-induced periodontitis than Lewis rats (Semenoff-Segundo et al., 2014). Moreover, Lewis rats serve as a classical model of a hyporesponsive HPA-axis (Oitzl et al., 1995) and there is a shift in balance between mineralocorticoid and glucocorticoid receptors in brains of Lewis rats when compared to Wistar rats (Oitzl et al., 1995). The presence of mineralocorticoid and glucocorticoid receptors has also been determined in the inner ear and they likely contribute to the auditory physiology. In recent experiments, the expression of glucocorticoid receptors was proved essential for proper physiological hearing thresholds, whereas dowregulation of glucocorticoid receptors was connected with elevated thresholds and decreased hearing abilities (Heinrich et al., 2016). Thus, if differences among the two rat strains in the expression of those receptors can be confirmed, they may partly mediate different response of Wistar and Lewis rats to auditory stress. TNF-alpha is an inflammatory cytokine produced and released by many cell types. Elevated concentration of circulating TNF-alpha was detected in individuals with rheumatoid arthritis (Motivala et al., 2008) and the concentration increased following emotional stress. Moreover, individuals affected by conditions known to be associated with stress such as clinical depression (Liu et al., 2017) anxiety, post-traumatic stress disorder, and obsessive-compulsive disorder (Furtado and Katzman, 2015) also had elevated concentrations of TNF-alpha in blood. The higher concentration of TNF-alpha in serum of Lewis rats as compared to Wistar may be explained by a general tendency of Lewis rats to develop a proinflammatory profile (Perretti et al., 1993).

Differences between the rat strains regarding the corticosterone peak following acute stress were described in the literature using Lewis and Fischer rats as examples (Groeneweg et al., 2011). The elevated corticosterone was measured in Lewis rats 30 min after acute stress whereas in Fischer rats it was measured 10 min after stress. Others have not reported the late peak in corticosterone release seen by us in Lewis rats (2 weeks following stress) in rats. However, such peak was described in mice: adult male C57BL/6 mice subjected daily to 2 h of different types of stress (forced swim, restraint, etc.) had elevated corticosterone concentrations in the blood 15 days following stress (Bowers et al., 2008) as compared to baseline. Interestingly, the levels of corticosterone differed depending on the stressor used.

When establishing behavioral models, the age of the animals used needs to be taken into account. We used rats at an adolescent age (35–64 days). It has been shown that adolescent rats (Sprague Dawley) react to stress different from adult animals (Jankord et al., 2011). Depending on the developmental stage (early adolescence, late adolescence, adult), dramatic differences were seen in the reaction to chronic stress regarding tissue weight, body composition, and basal corticosterone levels, when tested in a stress model different from ours (Jankord et al., 2011).

In our previous and present experiments, we used only female animals. Although the animals were only 4 weeks old at the beginning of the experiments, 3 weeks later (end of the longest experiment) they could already be in the estrous cycle. Recent studies determined significant differences in hearing thresholds of women during different phases of the menstrual cycle. Interestingly, the lowest hearing thresholds correlated with the highest estrogen concentration (Souza et al., 2017). Whether serum estradiol correlates with hearing threshold in our paradigm is a matter of future studies.

The changes in levels of the four proteins examined in the auditory pathways of Wistar and Lewis rats differed, but those differences were moderate and did not correlate with differences between auditory function observed between the different time points after the cessation of the stress stimulus. It is thus unlikely that the plasticity-related genes mediate the differential auditory stress response. However, because of the way in which the samples were processed, we could not identify the cell types in which the changes occurred. Future experiments, for instance immunohistochemical analysis of brain slices obtained from stressed and unstressed animals, might provide evidence indicating which cells are involved in these changes. We demonstrated that AMPA receptor identified by pan-antibody that detects all four subunits of this fast synaptic transmission protein specific for glutamate is upregulated in Wistar rats 2 weeks following stress but downregulated in Lewis rats immediately following stress. However, the performance of auditory brain as per ABRs has not correlated with the changes observed. We have similar conclusions for other tested synaptic proteins, namely for Arc, Syt1, and Syt12. Although the statistically significant changes in the levels of these proteins were noted, at this point we cannot correlate the changes with functional performance of the auditory brainstem.

The fact that the emotional or social stress can affect functioning of cardiovascular, limbic, immune or gastrointestinal systems has been known for decades. Recent research has demonstrated that the same experimental stressor affects the behavior of different animal strains differently (Rex et al., 1996; Tang et al., 2005; Pecoraro et al., 2006; Kunze et al., 2014; Theilmann et al., 2016). Here, we demonstrate for the first time the evidence that not only the behavior, but also the auditory pathway of experimental animals may be affected by stress in a strain-dependent manner. This differential response is in that aspect reminiscent of human stress reactions, where some people respond to stress by auditory threshold increase, while others' hearing abilities gain sensitivity. Moreover, our results deliver some answers to the open question posed by Rauschecker and collaborators, regarding “*the factors influencing the resilience of some individuals against adverse circumstances, for example long-lasting stress, which can promote tinnitus (…) in others*” (Rauschecker et al., 2010, 2015). Here, we present for the first time strain-dependent differences of the auditory response to emotional stress, suggesting a possible role of genetic background as a factor influencing tinnitus resilience. Whether the two rat strains may thus provide two different models for testing therapeutic tinnitus interventions remains to be determined.

## Data availability statement

Datasets are available upon request.

## Authors contributions

AS, UR, HO, and BM planned and designed experiments. AS, UR, and OH performed the experiments and collected the data. AS, UR, OH, and GD analyzed the data. AS and GD wrote the main manuscript. All authors discussed the data and reviewed the manuscript.

### Conflict of interest statement

GD is employed by the company Dr. Willmar Schwabe GmbH & Co. KG a company involved in research, development, marketing and sales of plant-based medication. The remaining authors declare that the research was conducted in the absence of any commercial or financial relationships that could be construed as a potential conflict of interest. The reviewer LR and handling Editor declared their shared affiliation, at the time of review.
